# Bilateral Well Leg Compartment Syndrome as a Complication of Prolonged Lithotomy Position in Abdominoperineal Resection Surgery

**DOI:** 10.7759/cureus.17975

**Published:** 2021-09-14

**Authors:** Bing Howe Lee, Dashishka Thanuranga Wijetunga, Chitarth Rajasekaran, Clarissa Eng Su Min, Hamid Rahmatullah Bin Abd Razak

**Affiliations:** 1 Orthopaedic Surgery, Sengkang General Hospital, Singapore, SGP; 2 Physiotherapy, Sengkang General Hospital, Singapore, SGP; 3 Musculoskeletal Sciences, Duke-National University of Singapore (NUS) Medical School, Singapore, SGP

**Keywords:** well leg compartment syndrome, compartment syndrome, fasciotomy, lithotomy, foot drop

## Abstract

Patients undergoing prolonged surgery in a lithotomy position may develop acute lower limb compartment syndrome in the absence of trauma or pre-existing vascular disease, otherwise known as well-leg compartment syndrome (WLCS). Early recognition and management would prevent the potential, lethal complications associated with this condition. We present a case of a 55-year-old gentleman who developed bilateral WLCS after prolonged abdominoperineal resection of his pelvic liposarcoma.

## Introduction

Patients undergoing prolonged surgery in a lithotomy position may develop acute lower limb compartment syndrome in the absence of trauma or pre-existing vascular disease, otherwise known as well-leg compartment syndrome (WLCS). This can lead to significant postoperative morbidity and mortality. The incidence of this condition ranges from 0.20% to 0.03% [1]. Due to the rarity of this condition, there is a general lack of awareness as to its clinical course and management strategy. Early recognition and management would prevent potentially devastating complications. We present a case of a patient who developed WLCS after prolonged abdominoperineal resection of his pelvic liposarcoma.

## Case presentation

A 55-year-old Malay gentleman who was recently diagnosed with pelvic liposarcoma had undergone open abdominoperineal resection, cystectomy, and conduit by a multidisciplinary surgical team involving colorectal surgeons, urologists, and vascular surgeons. Surgery was performed with the patient positioned in a Lloyd-Davies modified lithotomy position. The total duration of the operation was nearly nine hours; otherwise, the surgery was uneventful with no intraoperative or immediate postoperative complications.

Following surgery, the patient was transferred to the high dependency unit for monitoring. On the first postoperative day (POD) one, the patient complained of pain in both lower limbs. An ultrasound scan of the lower limbs was performed and was negative for deep vein thrombosis (DVT). On POD two, the patient complained of worsening severe pain over the bilateral calves, worse over the left leg. An urgent referral was made to the on-duty orthopedic surgery team for concerns of compartment syndrome.

On examination by the on-duty orthopedic surgeon, there was diffuse and tense swelling over the anterolateral aspect of bilateral calves with tenderness on palpation. Passive inversion of the foot elicited pain over the anterolateral leg. The patient was also unable to actively dorsiflex his ankle or big toe. Distal sensation and pulses were present. The posterior calves were supple and non-tender. There was no pain on dorsiflexion of the foot. The patient was diagnosed to have bilateral well leg compartment syndrome (WLCS) of the anterior and lateral calf compartments, and a decision was made for an urgent bilateral leg fasciotomy.

As the left leg appeared to be clinically more advanced, a dual-incision four-compartment release was performed. For the right leg, a single-incision four-compartment release was performed instead. Intraoperatively, the anterior and lateral compartments of both calves were tight with bulging muscles. Serous fluid was noted upon release of the compartments and the muscles in the anterior and lateral compartments looked pale, especially in the right leg. Superficial and deep posterior compartments were not tense and had healthy-looking muscles.

Relook debridement was performed on POD four, which showed unhealthy muscles in the anterior and lateral compartments of both calves, worse on the left. On POD seven, the muscles were healthy and secondary closure was performed. The patient’s wound was inspected regularly and the sutures were removed two weeks after closure.

Postoperative rehabilitation

**Figure 1:**
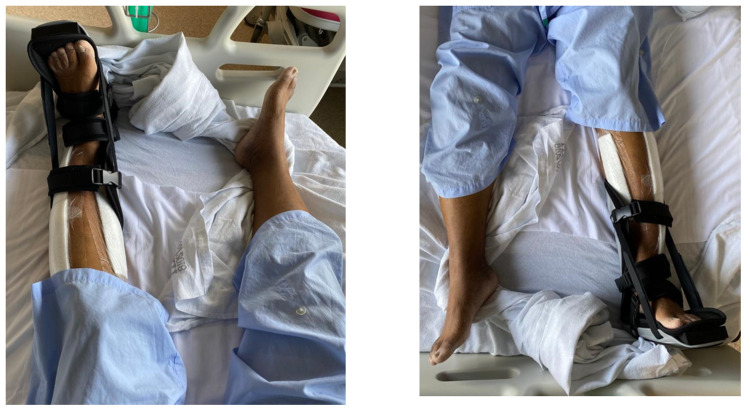
Left Ankle Foot Orthosis for Foot Drop The patient required the use of an ankle-foot orthosis on the left due to a foot drop from nerve damage.

The patient underwent a two-week period of intensive inpatient postoperative rehabilitation, focusing on a combination of progressive strengthening, balance, and gait practice. He required a static ankle-foot orthosis (AFO) on his left due to a foot drop (Figure 1). He also had diminished sensation over bilateral lower limbs. By POD 14, he was able to participate in three physiotherapy sessions per day, demonstrated significant improvements in functional mobility, and was able to ambulate using a broad-based quad stick.

The patient was discharged home on POD 22. At four months post-surgery, he was able to ambulate independently with no walking aid within his house and used a broad-base quad stick with the static left AFO in the community.

## Discussion

We report the case of a patient who developed bilateral WLCS after undergoing prolonged pelvic surgery in the lithotomy position. He required urgent fasciotomy and multiple relook surgical debridements, eventually resulting in significant morbidity and a left foot drop.

The development of WLCS is a potentially devasting complication of prolonged pelvic surgery. There have been multiple published case reports of this condition, yet it remains under-recognized [2]. This might be related to the rarity of WLCS, with a reported incidence of 0.03 to 0.20% [1]. However, it is associated with significant morbidity, with the incidence of permanent disability after WLCS ranging from 30% to 100% [2-3].

The mechanism of WLCS is primarily related to the ischemia-reperfusion of the lower limbs after placement in a prolonged lithotomy position for ≥ four hours [4-5]. Elevation of the lower limbs results in a fall in the mean arterial pressure in the calves. This is compounded by the increase in compartment pressures associated with limb elevation and can result in significant and unrecognized intraoperative lower limb ischemia. A reduced venous return associated with the raised intra-abdominal pressure accompanying laparoscopic/robotic surgery, intraoperative hypotension, and the use of vasoconstrictors associated with epidurals are additional contributors. This leads to tissue edema within the lower limb compartments and marked intracompartmental hypertension. This is further aggravated once the legs are lowered and reperfusion of ischemic muscles occurs.

Patients affected by WLCS typically complain of severe calf pain after surgery, within 24 hours of recovery from anesthesia [6]. They exhibit the characteristic symptoms and signs of compartment syndrome (unremitting pain, worse with passive stretch of muscles within that compartment, pallor, cold, paraesthesia, foot drop, and vascular insufficiency in the late stage). Diagnosis is based on clinical judgment, and urgent four-compartment fasciotomy is warranted.

There are published multidisciplinary clinical guidelines for the prevention, diagnosis, and management of WLCS [4,7]. Table 1 summarizes the key recommendations, and they should be adhered to in all prolonged abdominopelvic surgeries in lithotomy positions.

**Table 1:** Key Recommendations from the United Kingdom and Ireland Multidisciplinary Clinical Guidelines on Well Leg Compartment Syndrome Source: [4]

No.	Key Recommendations
1.	All surgeons who undertake pelvic procedures on patients maintained in the Lloyd-Davies/lithotomy positions should be aware of well leg compartment syndrome (WLCS).
2.	The risk of WLCS must be noted specifically in the preoperative team brief and WHO time out. Strategies agreed to minimize the risk to each patient must be recorded before the commencement of surgery.
3.	Unless mandated by other patient safety considerations, the patient’s legs should be kept at a level below the heart for the maximum duration possible during a procedure.
4.	Where elevation of the legs is required to facilitate surgery, the maximum unbroken period of elevation should not exceed four hours. The patient’s legs should be kept at a lower level than the heart for a minimum of 15 min after each four-hour interval. The duration of elevation and the time allowed for recovery should be monitored and documented in the patient’s operation note/anesthetic chart.
5.	Intraoperative hypotension should be corrected where possible and intraoperative fluid therapy optimized to avoid both excessive fluid administration and inadequate tissue perfusion.
6.	Any patient who has undergone pelvic surgery in the lithotomy position, whether or not combined with Trendelenberg tilt, who complains of postoperative leg pain should be suspected of having WLCS.
7.	The initial diagnosis of WLCS is entirely clinical, so assessment of the patient with suspected WLCS must be methodical, focused, thorough, and documented clearly and contemporaneously.
8.	Assessment must include: a. Accurate history – pain, paraesthesia, numbness, weakness, paralysis. b. Pain – onset, site, nature. c. Presence of paraesthesia or numbness. d. Inspection: swelling – unilateral/bilateral, edema. e. Palpation: tension in compartments, palpable difference between sides; tenderness in each compartment. f. Passive stretch exacerbation of pain: dorsiflexion of toes, plantar flexion of toes, dorsiflexion/plantarflexion of ankle. g. Assessment of sensation, pulses, and capillary refill.
9.	If WLCS is suspected, immediate referral should be made to the orthopedic or vascular surgery team, according to local protocols.
10.	If clinical assessment confirms a definite or likely diagnosis of WLCS, this is a limb/life-threatening surgical emergency requiring immediate decompression by open four-compartment fasciotomy. Treatment should be underway within 1 h of diagnosis.
11.	There should be a low threshold for reassessment in patients whose symptoms persist or deteriorate.
12.	If initial assessment is equivocal, for example, in a sedated/unconscious patient, in whom adequate clinical assessment cannot be undertaken, and there is a high index of suspicion, measurement of compartment pressures may be used to confirm or exclude compartment syndrome.
13.	If compartment pressure measurement is undertaken, and the difference between diastolic pressure and compartment pressure is less than 30 mmHg in any compartment, an immediate (within 1 h), bilateral four-compartment fasciotomy should be undertaken.
14.	Reassessment of the compartments in the theater should be carried out between 48 and 72 h after decompression.
15.	After fasciotomy, early discussion with, and involvement of, plastic and reconstructive surgeons is recommended for patients with significant tissue loss.

In this case, the patient's limbs were elevated for more than four hours, without any interval lowering. A DVT ultrasound scan was done on POD one, which potentially delayed the recognition of WLCS. This resulted in significant morbidity for the patient, who has permanent nerve and muscle damage and a resultant foot drop.

Delay in the recognition and treatment of compartment syndrome can lead to the development of nerve injury [8]. The superficial peroneal nerve (SPN) and deep peroneal nerve (DPN) are the most commonly affected and can result in numbness in the dorsum of the foot and a foot drop, respectively. Lollo et al. reported that the severity of nerve injury worsens with the delay in performing fasciotomy, with more patients developing foot drop from a DPN injury [9] as compared to an SPN injury.

## Conclusions

Well leg compartment syndrome (WLCS) is not a common condition, but it is devastating for patients who develop it. Clinicians should adhere to the guidelines on the prevention of WLCS during prolonged pelvic surgery in the lithotomy position and maintain a high index of suspicion postoperatively. Early recognition and fasciotomy is key to prevent significant morbidity.
